# Genome Sequence of an Unusual Reassortant H1N1 Swine Influenza Virus Isolated from a Pig in Russia, 2016

**DOI:** 10.1128/genomeA.00747-17

**Published:** 2017-09-07

**Authors:** Ivan Sobolev, Olga Kurskaya, Tatyana Murashkina, Sergey Leonov, Kirill Sharshov, Marsel Kabilov, Tatyana Alikina, Natalia Tolstykh, Vladimir Gorodov, Alexander Alekseev, Alexander Shestopalov

**Affiliations:** a Research Institute of Experimental and Clinical Medicine, Novosibirsk, Russia; b Institute of Experimental Veterinary Science of Siberia and the Far East, Siberian Federal Scientific Centre of Agro-BioTechnologies, Krasnoobsk, Russia; c Institute of Chemical Biology and Fundamental Medicine SB RAS, Novosibirsk, Russia

## Abstract

We report here the genome sequence of the influenza A virus strain A/swine/Siberia/1sw/2016, isolated from a swine in Russia. On the basis of sequence analysis, A/swine/Siberia/1sw/2016 is characterized by unusual surface glycoproteins phylogenetically distinct from those of swine A(H1N1)pdm09 influenza virus.

## GENOME ANNOUNCEMENT

Swine influenza virus causes an acute and highly contagious respiratory disease in swine. The pig can be a mixing vessel for generating new genetic variants of influenza virus (1–3) because the cells of the swine respiratory tract contain receptors preferred by both human (α-2-6-linked sialic acid) and avian (α-2-3-linked sialic acid) influenza viruses. In particular, the influenza A(H1N1)pdm09 virus that emerged in the human population in 2009 was derived from pigs, and its genome contained human, swine, and avian virus genes (4, 5).

Here, we report the genome sequence of influenza A virus strain A/swine/Siberia/1sw/2016, initially isolated from a swine pulmonary tissue sample. Sectional material from pigs was collected on a private pig farm in Russia in 2016. Influenza virus A was detected by real-time PCR. The strain was isolated on Madin-Darby canine kidney (MDCK) cells. Complete genome sequencing was performed in the SB RAS Genomics Core Facility (ICBFM SB RAS, Novosibirsk, Russia). Viral RNA was extracted using TRIzol LS (Invitrogen, USA). cDNA was synthesized using a random hexamer primer with RevertAid Premium (Thermo Scientific, USA). The second DNA chain was synthesized using the NEBNext mRNA second-strand synthesis module (NEB, USA), in accordance with the manufacturer’s instructions. DNA libraries were made using TruSeq DNA sample prep kits (version 2), as specified by the manufacturer (Illumina, USA). The DNA sequence of the libraries was made using the MiSeq system (Illumina, USA) with version 2 reagent kits (300 paired-end), according to the manufacturer’s instructions. Sequence assembling was conducted using the CLC Genomics Workbench version 5.5 software (CLC bio, USA).

The genome of the A/swine/Siberia/1sw/2016 strain consisted of the following eight gene segments: polymerase (PB2, PB1, and PA), hemagglutinin (HA), nucleoprotein (NP), neuraminidase (NA), matrix protein (M), and nonstructural protein (NS) genes. The sequence of the PB2 gene consisted of 2,195 nucleotides (nt), the PB1 gene 2,328 nt, the PA gene 1,965 nt, the HA gene 1,776 nt, the NP gene 1,551 nt, the NA gene 1,405 nt, the M gene 1,015 nt, and the NS gene 890 nt.

Phylogenetic analysis showed that genes encoding surface glycoproteins (HA and NA) of the A/swine/Siberia/1sw/2016 strain are significantly different from the HA and NA gene sequences represented in databases (GenBank, Global Initiative on Sharing All Influenza Data [GISAID], and Influenza Research Database [IRD]). The closest (90%) sequences of HA and NA belong to the influenza A (H1N1) virus isolated from humans in the United States in the 1980s. Thus, the phylogenetic strain A/swine/Siberia/1sw/2016 refers to the subtype H1N1, but it is not a modern pandemic variant of A(H1N1)pdm09. However, the nucleotide sequences of the genes encoding the internal proteins of the virus (PB2, PB1, PA, NP, MP, and NS) are similar to the genes of the influenza A(H1N1)pdm09 virus isolated from humans.

Thus, the A/swine/Siberia/1sw/2016 influenza virus strain is a previously unregistered locally circulating reassortant variant of the H1N1 subtype in the pig population.

### Accession number(s).

The genome sequence of A/swine/Siberia/1sw/2016(H1N1) was deposited in GenBank under the accession numbers MF370340 to MF370347.
